# Cultivation of enteroids from fresh and cryopreserved bovine duodenal tissues

**DOI:** 10.3168/jdsc.2023-0379

**Published:** 2023-08-19

**Authors:** Koki Nishihara, Katie M. Wood, Le Luo Guan, Michael A. Steele

**Affiliations:** 1 Department of Animal Biosciences, Animal Science and Nutrition, University of Guelph, Guelph, ON, Canada N1G 1Y2; 2 Department of Agricultural, Food and Nutritional Science, University of Alberta, Edmonton, AB, Canada T6G 2P5

## Abstract

This study aimed to develop a method for intestinal tissue cryopreservation and resuscitation for enteroid cultivation. Two different types of tissues, fresh duodenal tissues (n = 3, from Angus steers) and duodenal tissues cryopreserved in 90% fetal bovine serum (FBS) and 10% dimethyl sulfoxide (DMSO; n = 3, from Holstein calves), were collected to develop enteroids. Crypts were isolated using 2 m*M* EDTA/phosphate-buffered saline from both fresh and cryopreserved tissues and embedded in basement membrane extract. Embedded crypts were seeded in a 24-well plate and cultured in IntestiCult Organoid Growth Medium (Mouse) with inhibitors cocktail and Primocin. The upper opening of crypts became sealed, and crypts formed sphere structures (i.e., enteroids) within 24 h. Primary (passage 0) enteroids showed budding crypt domains from d 3 of cultivation at the earliest. After 7 d of cultivation, enteroids were passaged in a new 24-well plate. Fragments from passaged d 7 enteroids also formed sphere structures within 24 h after seeding and showed budding crypt domains from d 3 of cultivation at the earliest. The area of enteroids was measured in each animal during d 1 to 7 in passage 0 and 1, and the area of enteroids derived from both tissues increased during d 1 to 7 in passage 0 and 1. The area increased from d 1 to 7 of cultivation, and the area of passage 1 was greater than that of passage 0. F-actin staining using phalloidin revealed that brush border microvilli were distributed on the luminal side of the enteroids. In conclusion, a cryopreserved solution consisting of FBS and DMSO is useful for cryopreservation and resuscitation of bovine intestine for enteroid cultivation. This method allows researchers to investigate intestinal function and health in the laboratory using enteroids derived from fresh and cryopreserved tissues collected from cattle.

## Highlights

• Tissues were cryopreserved in fetal bovine serum and dimethyl sulfoxide.
• Cryopreserved tissues could be resuscitated for enteroid development.
• Enteroids derived from cryopreserved tissues were reseeded and cultivated.

## Graphical Abstract

**Figure:**
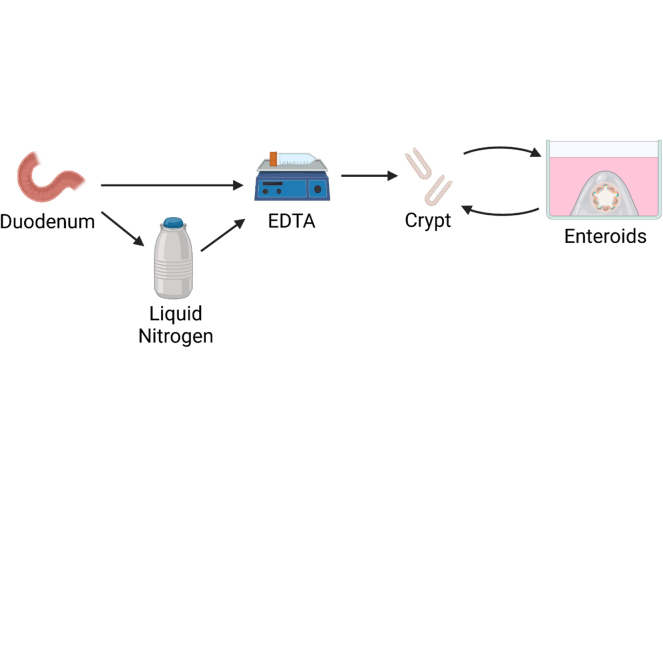
**Summary:** In case fresh tissues are not available for crypt isolation from the intestine immediately after tissue collection, developing a method for tissue cryopreservation would help enteroid formation, an important in vitro model. This technical note describes an established method for intestinal tissue cryopreservation and resuscitation for cattle, as well as bovine enteroid cultivation. Fetal bovine serum (90%) and dimethyl sulfoxide (10%) were used as a tissue cryopreservation solution, and tissues were cryopreserved until use. Crypts were isolated from fresh and cryopreserved tissues of cattle using 2 mM ethylenediaminetetraacetic acid/phosphate-buffered saline, and isolated crypts embedded in basement membrane extract were seeded in a 24-well plate and formed enteroids within 24 hours. After 7 days of cultivation, fragments derived from enteroids from both fresh and cryopreserved tissues were reseeded in a new 24-well plate and were able to be cultivated for 7 days. This tissue cryopreservation method enables researchers to establish stable bovine enteroids to investigate intestinal functions.

Intestinal functions are key to calf and cow performance. To better understand the function of the small intestine, animal models (i.e., in vivo models) are regularly used in dairy and beef science. However, in vivo models are time consuming and human resource intensive to conduct and bring about ethical issues, as some studies may require the slaughter of animals to obtain tissue samples. While in vitro models, such as immortalized cell lines (e.g., Caco-2), which can be cryopreserved and still proliferate, do not fully represent the interactions between multiple cell types, they are commonly used in medical science to compensate for the drawbacks of in vivo models. However, as immortalized bovine intestinal cell lines have not been established (Beaumont et al., 2021), in vitro models for bovine intestines are not widely used in dairy and beef science fields.

Organoids are one of the in vitro models used in the medical science field to study patient pathology, host-microbe interactions, drug toxicity, and tissue growth and homeostasis (Hofer and Lutolf, 2021). Enteroids, which are organoids derived from the small intestine, have 3-dimensional structures and multiple cell populations similar to those of the small intestine (Sato et al., 2009). Additionally, they display interactions between multiple cell types and near-physiological cellular composition and behaviors to traditional 2-dimensional cultures (Li and Izpisua Belmonte, 2019). Enteroids are used as models for analyzing host-microbial interactions (Zhang et al., 2014), patient pathology (Schwank et al., 2013), nutrient uptake (Co et al., 2019), and barrier integrity (Co et al., 2019) in medical science. Although bovine enteroids have been developed (Powell and Behnke, 2017; Hamilton et al., 2018; Derricott et al., 2019), adoption of this method in the research field is very limited. One of the reasons why bovine enteroids are not used in many laboratories is the lack of detailed methodologies in the literature. For example, how to handle the extracellular matrix hydrogel and cell signaling inhibitors used for enteroid cultivation has not been adequately described in the literature and therefore it is difficult to reproduce this methodology. Another reason may be the difficulty in obtaining fresh tissues because the dissection facility in most research farms is geographically far from the cell culture bench. The development of a methodology for tissue cryopreservation and resuscitation (e.g., Tsai et al., 2018) may help overcome this limitation, as it would increase the flexibility of research by eliminating the dependence on time and place. In addition, long-term storage allows for future reuse, which could help to reduce the number of animals used in animal experiments. Therefore, the objective of this study was to develop a method for intestinal tissue cryopreservation and resuscitation from bovine tissues, as well as for enteroid cultivation. We hypothesized that a traditional solution for cell cryopreservation could be used for tissue cryopreservation in the development of enteroids.

The washing buffer, cryopreservation solution, digestive solution, primary medium, growth medium, and B-27 Advanced DMEM/F12 used in this study are listed in Table 1. Cultrex Reduced Growth Factor Basement Membrane Extract, Type 2 (**BME**, 3533–010–02, Bio-techne, USA) was aliquoted into 1.5-mL tubes on ice and then stored at −20°C until use.

**Table 1:** Reagents used in establishment of bovine enteroids

Reagent name	Supplier	Catalog no.	Solvent	Stock concentration	Final concentration
Washing buffer					
PBS^1^	Sigma-Aldrich	P5368	H_2_O	—	—
Fetal bovine serum^2^	Thermo Fisher Scientific	FB12999102	—	—	5% (vol/vol)
Penicillin/streptomycin^3^	Thermo Fisher Scientific	15140122	—	10,000 IU/mL	100 IU/mL
Gentamicin^4^	Thermo Fisher Scientific	15750060	—	50 mg/mL	25 μg/mL
Cryopreservation solution			—		
Fetal bovine serum^2^	Thermo Fisher Scientific	FB12999102	—	—	—
Dimethyl sulfoxide^5^	Thermo Fisher Scientific	AAJ66650AE	—	—	10% (vol/vol)
Digestive solution					
PBS^1^	Sigma-Aldrich	P5368	H_2_O	—	—
EDTA^1^	Thermo Fisher Scientific	AAJ15694AE	PBS	0.5 *M*	2 m*M*
Primary medium^6^					
IntestiCult Organoid Growth Medium (Mouse)^7^	STEMCELL Technologies	6005	—	—	—
Y-27632 (dihydrochloride)^8^	STEMCELL Technologies	72304	—	50 m*M*	10 μ*M*
Galunisertib (LY2157299)^8^	Selleck	S2230F	DMSO	50 m*M*	500 n*M*
FHPI (SB202190)^8^	Selleck	S1077	DMSO	100 m*M*	10 μ*M*
Primocin^9^	InvivoGen	ant-pm-1	—	50 mg/mL	100 μg/mL
Growth medium^6^, ^10^					
B-27 Advanced DMEM/F12^6^					
Advanced DMEM/F12^9^	Thermo Fisher Scientific	12634010	—	—	—
Gibco B-27 supplement (50×), minus vitamin A^11^	Thermo Fisher Scientific	12587010	—	50×	1×
Primocin^9^	InvivoGen	ant-pm-1	—	50 mg/mL	100 μg/mL

1 Autoclaved.

2 Preheated to 56°C for 30 min and stored at −20°C until use.

3 Aliquoted to 15-mL tubes on ice and then stored at −20°C.

4 Aliquoted to 1.5-mL tubes on ice and then stored at −20°C.

5 Sterlized by filtration.

6 Used within 2 wk after preparation.

7 Aliquoted into 15-mL tubes and stored at −20°C.

8 Aliquoted to 200-μL tubes on ice and then stored at −20°C.

9 Aliquoted to 1.5-mL tubes on ice and then stored at −20°C.

10 Prepared in the same way as primary medium except for Y-27632.

11 Aliquoted in a 1.5-mL tube and stored at −20°C, protected from light.

A total of 6 animals (3 Angus steers and 3 Holstein bull calves) were used in this study. Three Angus steers were born and raised at the Ontario Beef Research Station. Angus steers were raised in accordance with the Canadian Council on Animal Care (Olfert et al., 1993), and all procedures were approved by the Animal Care Committee (University of Guelph protocol #4310). After weaning, Angus calves were housed in grouped feedlot pens (12 cattle/pen) and fed finishing diets consisting of 57% high-moisture corn, 20% cracked corn, 12% alfalfa haylage, 9.5% soybean meal, and 1.5% vitamins/minerals/supplements on a DM basis, until they reached finishing weight (692 ± 27 kg). All diets contained 33 mg/kg monensin within the vitamin/mineral premix. Angus steers were slaughtered at the University of Guelph Meat Laboratory (Guelph, Canada). Steers (~15 mo old) were euthanized by captive bolt and exsanguination. The duodenal tissues were obtained 1 h after exsanguination. The site at which the duodenum crosses to the transverse colon was collected and kept in ice-cold PBS. The collected tissues were brought to the cell culture hood within 5 min after tissue collection. Additionally, 3 intact male Holstein calves were obtained from a commercial dairy farm and housed in individual pens at the Ponsonby general animal facility at the University of Guelph (Guelph, Canada). All procedures were approved by the Animal Care Committee (University of Guelph protocol #4470). Calves were fed a texturized starter ad libitum (40% starch, 18% CP, and 20% NDF; Surgain, ON, Canada) until 42 d of age. The calves were euthanized by captive bolt and exsanguination. The duodenal tissues were obtained within 30 min after exsanguination. The duodenal tissues were kept in ice-cold PBS during transportation to the cell culture hood within 30 to 60 min.

After tissues were brought into the laboratory, they were placed in a 10-cm dish with ice-cold washing buffer. The connective tissues of the duodenum were removed as much as possible, and the duodenal tissues were cut into small pieces (approximately 1 cm^2^) and transferred into a 50-mL tube. Tissues were suspended in 10-mL ice-cold washing buffer, and the 50-mL tube was vortexed on high speed for 10 s. The tube was placed on ice until the tissue settled by gravity. The washing buffer was removed by an aspirator. This washing procedure was repeated until the supernatant was clear (approximately 10 times). The duodenal tissues obtained from Angus steers were used for further crypt isolation. Only duodenal tissues obtained from Holstein calves were used for cryopreservation.

A piece of the duodenal tissue (approximately 1 cm^2^) obtained from Holstein calves was placed in a cryovial and resuspended in 1 mL of ice-cold cryopreservation solution. Cryovials were placed overnighted in a freezing container (Nalgine Mr. Frosty, N51000001, Thermo Fisher Scientific), stored at −80°C overnight, transferred to −155°C the following day, and stored until further crypt isolation. To resuscitate the tissues, cryovials were soaked in water (37°C) for 1 min, and the cryopreservation solution was removed. Resuscitated tissues were washed in ice-cold washing buffer 3 times before crypt isolation in a 10-cm dish. All cryopreserved tissues were resuscitated within 4 mo.

The procedures of crypt isolation and embedding in BME are illustrated in Figure 1. To isolate crypts from fresh tissues (Angus steers) and cryopreserved tissues (Holstein calves), 8 to 16 pieces of tissues were resuspended in 25 mL of ice-cold digestive solution in a 50-mL tube. The tube was incubated with constant, gentle shaking for 30 min on ice. After shaking, tissues were settled by gravity for 60 s on ice, 20 mL of supernatant was removed, and 5 mL of supernatant was left to cover the tissues. Then, 10 mL of ice-cold washing buffer was added to the tube (total volume was 15 mL), and the tube was vortexed at high speed for 10 s to release additional crypts. Tissues were settled by gravity for 30 s on ice, and 10 mL of supernatant was filtered through a 100-μm cell strainer into a new 50-mL tube to remove the villus fraction and to collect crypt fractions. The resuspension, vortexing, and filtration steps were repeated 4 times total. A total of 50 µL of the supernatant was collected from each tube and applied to a glass slide for further crypt counting. The 4 tubes were centrifuged at 200 × *g* for 5 min at 4°C. During centrifugation, the number of crypts was counted for each tube. One or 2 tubes with the highest number of crypts were selected for further analysis, and the supernatant was discarded after centrifugation. The crypts were resuspended in 10 mL of Advanced DMEM/F12 to remove FBS and centrifuged at 200 × *g* for 5 min at 4°C. The supernatant was discarded and the crypts were resuspended in ice-cold B-27 Advanced DMEM/F12 at a concentration of 1,000 crypts/100 μL of medium. Then, 100 μL of this solution was added to 150 μL of ice-cold BME on ice. Using the same tip, this mixture was pipetted gently, and 50 μL of the droplet was added to the center of each well in a 24-well plate that had been previously warmed at 37°C for at least 30 min. The 24-well plate was then incubated at 37°C in a 5% CO_2_/air atmosphere for 15 min to solidify BME. After 15 min, 650 μL of pre-warmed primary medium was slowly pipetted down the sidewall of each well, and the 24-well plate was returned to the incubator. After 24 h, the medium was replaced with 650 μL of growth medium and the 24-well plate was returned to the incubator. Three days after medium replacement, the medium was replaced with growth medium. The 24-well plate was incubated for a total of 7 d.

**Figure 1:**
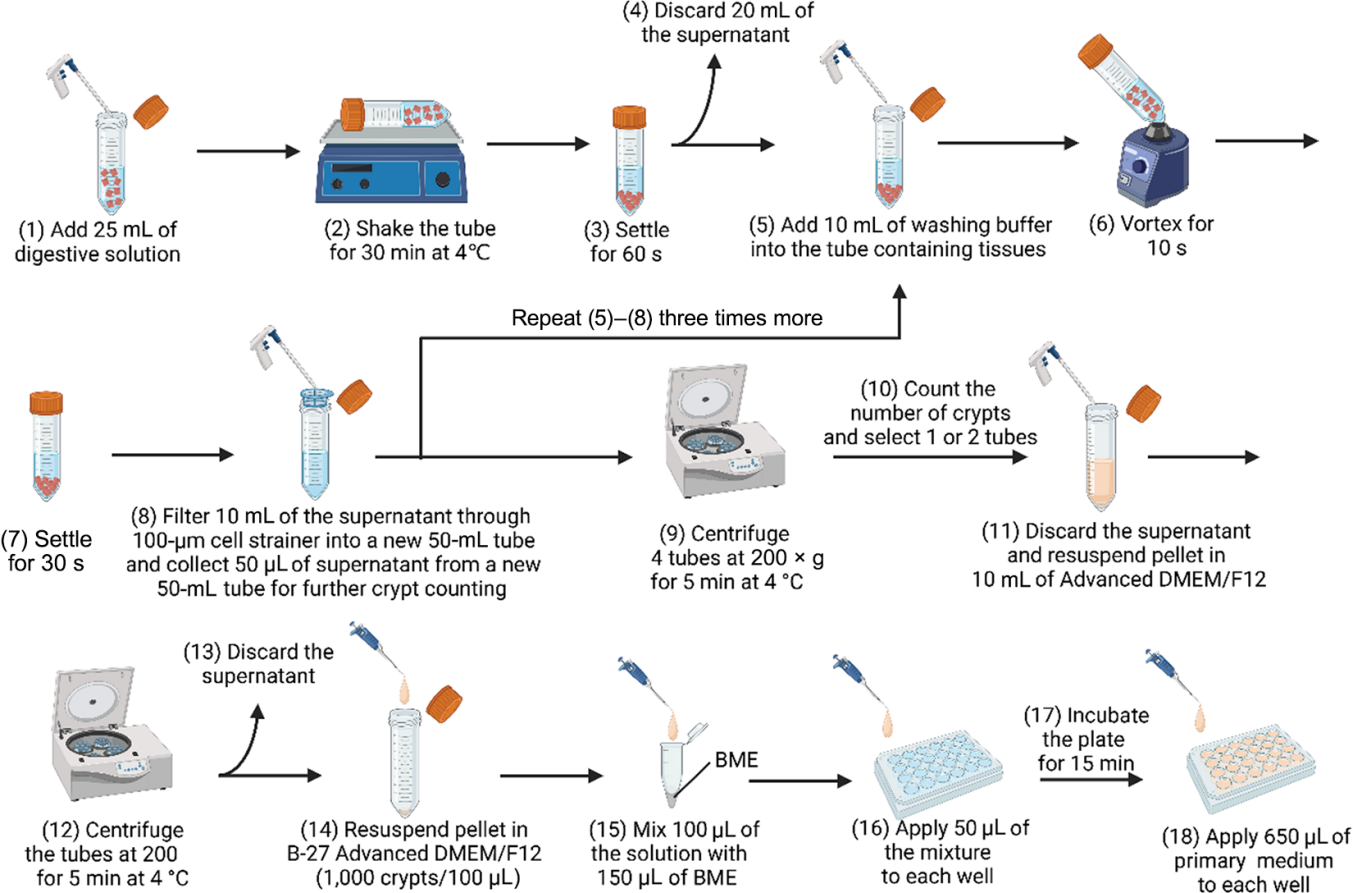
Isolation and embedding of the crypts were performed according to methods (1) through (18). Procedures (5) to (8) were repeated until 4 filtered 50-mL tubes were obtained. Procedures (1) to (15) were done on ice except for vortexing and centrifugation. This illustration was created using BioRender.com.

After 7 d of cultivation, the medium was removed without disturbing the dome of the enteroids in the BME. Then, 1 mL of ice-cold TrypLE (12604–021, Thermo Fisher Scientific) was added to each well. The solution was pipetted 20 times to break the BME dome, and the solution was transferred to a 15-mL tube. The 15-mL tube was incubated at room temperature (**RT**) for 8 min and gently pipetted 10 times every 2 min. After 8 min, Advanced DMEM/F12 was added until the total volume reached 10 mL, and the number of fragments in 50 μL of the solution was then counted. The tube was then centrifuged at 300 × *g* for 5 min at 4°C, and the supernatant was discarded. The concentration was adjusted to 1,000 fragments/100 μL of ice-cold B-27 Advanced DMEM/F12. This solution was mixed with BME, and 50 μL was added to each well in a 24-well plate that had been warmed at 37°C for at least 15 min. After 15 min, 650 μL of pre-warmed primary medium was slowly pipetted down the sidewall of each well, and the 24-well plate was returned to the incubator. After 24 h, the medium was replaced with 650 μL of growth medium, and the 24-well plate was returned to the incubator. Three days after medium replacement, the medium was replaced with growth medium. The 24-well plate was incubated for a total of 7 d.

Texas Red-conjugated phalloidin (T7471, Thermo Fisher Scientific) was used to stain F-actin in the brush border microvilli of enteroids. The medium from the 24-well plate was removed without disturbing the dome of organoids in the BME, and 1 mL of ice-cold PBS was added on top of the exposed dome in each well to dissolve BME. Enteroids were slowly transferred to 1.5-mL microcentrifuge LoBind tubes (698–794, Thermo Fisher Scientific) using a pipette and pelleted at 300 × *g* for 1 min at 4°C. Enteroids were incubated in 1 mL of paraformaldehyde fixative solution (2% [wt/vol] paraformaldehyde [AA473479L, Thermo Fisher Scientific], 60 m*M* dibasic sodium phosphate [567550500GM, Thermo Fisher Scientific], and 14 m*M* monobasic sodium phosphate [AC448170010, Thermo Fisher Scientific] in water) for 30 min at RT. Enteroids were washed with 1 mL of ice-cold PBS and centrifuged at 300 × *g* for 1 min at 4°C. This procedure was repeated 3 times, after which the enteroids were incubated with blocking/permeabilization buffer (3% [wt/vol] bovine serum albumin [A7906, Sigma-Aldrich], 1% [wt/vol] saponin [AAA1882014, Thermo Fisher Scientific], 0.02% [wt/vol] sodium azide [AA1431422, Sigma-Aldrich, USA], and 1% [vol/vol] Triton-X [112298, Sigma-Aldrich]) at 4°C overnight. The enteroids were then washed with 1 mL of ice-cold PBS and centrifuged at 300 × *g* for 1 min at 4°C. This procedure was repeated 3 times, after which enteroids were incubated with 400 μL of Texas Red-conjugated phalloidin solution for 60 min at RT. Enteroids were washed with 1 mL of ice-cold PBS and spun at 300 × *g* for 1 min at 4°C. This wash procedure was repeated 3 times, after which they were incubated with DAPI (5087410001, Sigma-Aldrich) solution (diluted at 1:500) for 4 h at RT. Enteroids were washed with 1 mL of ice-cold PBS and centrifuged at 300 × *g* for 1 min at 4°C. This procedure was repeated 3 times, then enteroids were resuspended in 1 droplet of ProLong Diamond Antifade Mountant (P36965, Thermo Fisher Scientific). Enteroids were then transferred to a glass slide, and the distribution of phalloidin was observed using Diskovery Spinning Disk (Leica, Germany) and imaged using Volocity (x64 software).

Bright field images of the enteroids were taken using Cytation5 (BioTek, USA). Images were taken at the same x, y, and z positions from d 1 to 7 to trace the same enteroids' morphology and area from each animal and passage. From 2 to 10 enteroids (i.e., technical replicates) could be traced from d 1 to 7 in each animal and passage. Enteroid area (μm^2^) in each animal, passage, and day was measured using a polygon tool in ImageJ Fiji software. If enteroids overlapped with other enteroids or were missing from the edge of the image at a given time point, the area of the enteroid at that time point was not measured and was considered missing. If missing data were surrounded by valid data, they were imputed by linear interpolation using the pandas library (v 1.3.5) in Python software (v 3.8.16) by setting limit_area as ‘inside.' During d 1 to 7, 11% (86/756; 15% [51/336] in fresh tissues group and 8% [35/420] in cryopreserved tissues group) of the data were missing and excluded from the average calculation. The average area for each animal and passage was calculated at each day and used for statistical analysis. The images of d 6 and 7 in passage 1 of 1 Angus steer and d 7 in passage 1 for 2 Holstein calves were missed. Therefore, the area of these cattle at these time points was excluded from the statistical analysis. Using lme4 (v 1.1.31) package in R software (v 4.1.3), area of enteroids derived from fresh and cryopreserved tissues was respectively analyzed by Generalized Linear Mixed Model. The model included day (1, 2, 3, 4, 5, 6, and 7) and passage times (0 and 1) as fixed effects, and calf as a random effect. Packages of lmerTest (v 3.1.3), car (v 3.1.1), and lsmeans (v 2.30.0) in R software were used for *P*-value calculation. Significance was declared at *P*-value <0.05.

Representative crypts obtained from the duodenal tissues using digestive solution are shown in Figure 2A. Representative bright field images of enteroids derived from fresh and cryopreserved tissues on each day and passage are shown in Figure 2B. The upper opening of crypts (i.e., the area opposite the lower part of the crypts where the stem cells are located) became sealed and the crypt formed a sphere structure (enteroid) within 24 h after seeding in BME (Figure 2B). Some crypts from all tissues in each well failed to form enteroids (data not shown). Enteroids showed budding crypt domains during cultivation (Figure 2C) on d 3 at the earliest (data not shown). The area of passage 1 was greater than that of passage 0 in both tissues type (*P* < 0.05). Phalloidin, which binds to F-actin in brush border microvilli, was distributed in the lumen of enteroids (Figure 2D). Fragments from passaged d 7 enteroids formed sphere structures again within 24 h after seeding in a new 24-well plate and showed budding crypt domains (Figure 2B). The areas of the enteroids in each animal are shown in Figure 2E and 2F. Seven days of cultivation revealed that the area increased from 1,454.9 ± 298.4 μm^2^ to 5,498.3 ± 1,461.0 μm^2^ (*P* < 0.05) in enteroids derived from fresh tissues and 1,520.6 ± 280.5 μm^2^ to 6,478.4 ± 1,690.2 μm^2^ (*P* < 0.05) in enteroids derived from cryopreserved tissues during d 1 to 7 of cultivation.

**Figure 2:**
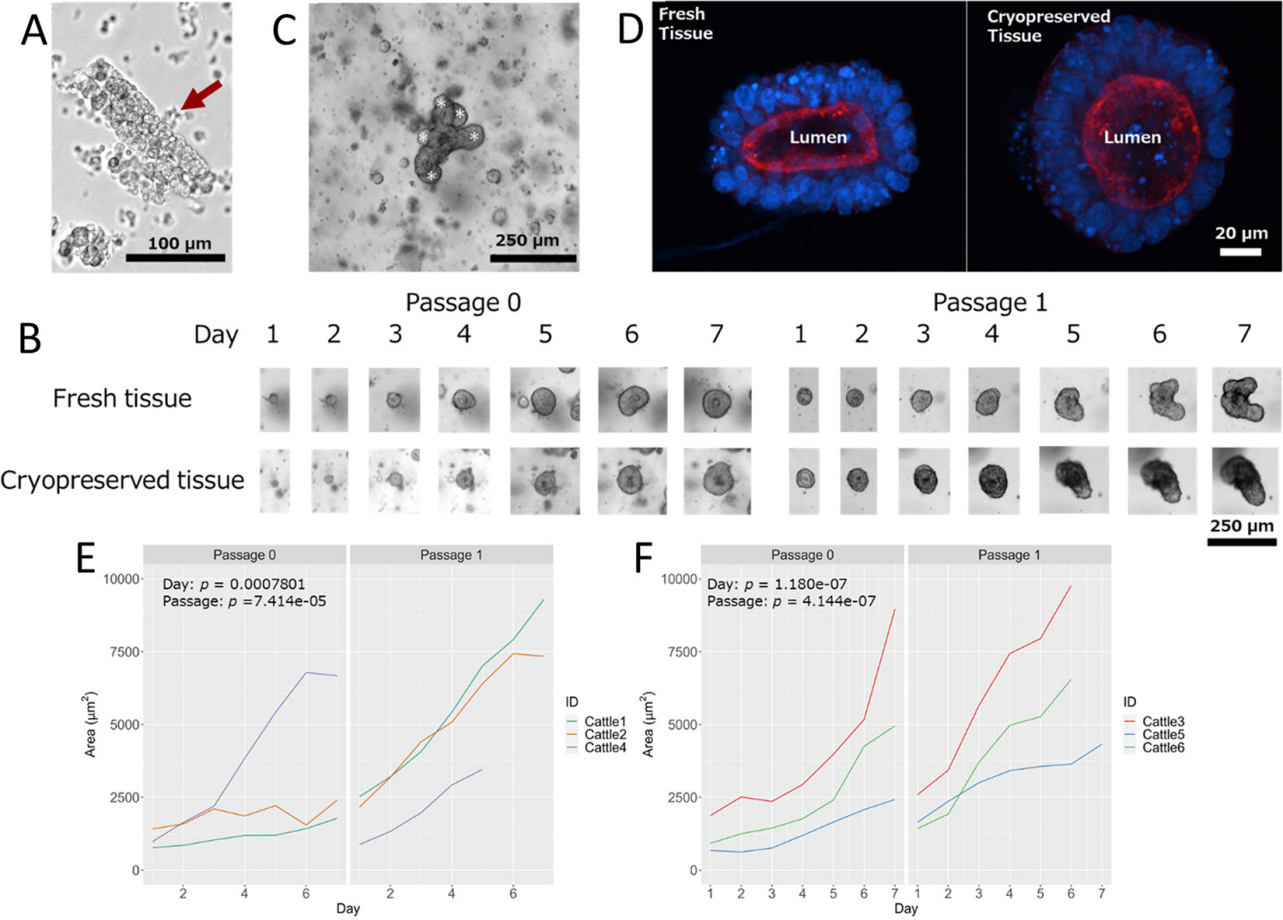
(A) Example of crypt isolated using digestive solution. Arrow (red) shows crypts obtained from the duodenal tissue. (B) Images of enteroids obtained from fresh tissue (Angus steer) and cryopreserved tissue (Holstein calf) from d 1 to 7 in passages 0 and 1. (C) Budding of crypt domains of enteroid on d 5. A white asterisk indicates a budding crypt domain. (D) Confocal images of enteroids (d 3 in passage 0 derived from fresh [left] and cryopreserved [right] tissue). Nuclei were stained by DAPI (blue; Sigma-Aldrich), and F-actin in the brush border microvilli was stained using Texas Red-conjugated phalloidin (red; Thermo Fisher Scientific). Enteroid area (μm^2^) in each calf and passage from d 1 to 7 from fresh tissues from Angus steers (n = 3 [n = 2 in passage 1]; E) and cryopreserved tissues from Holstein calves (n = 3 [n = 1 at d 7 in passage 1]; F).

A traditional cell cryopreservation solution consisting of 90% FBS and 10% DMSO is used in several cell lines (Loretz et al., 1989). As hypothesized, we succeeded in developing enteroids from tissues cryopreserved in 90% FBS and 10% DMSO solution. Culturing enteroids derived from cryopreserved tissues was similar to the procedure for enteroids derived from fresh tissues. Enteroids derived from both tissues showed similar morphology (i.e., budding crypt domains and F-actin distribution in the lumen side) and grew during d 1 to 7 in passages 0 and 1. This cryopreservation solution was also used to develop organoids from cryopreserved tissues (duodenum, ileum, and colon) in humans (Tsai et al., 2018). This suggests that 90% FBS and 10% DMSO could also be useful for the development of enteroids from cryopreserved bovine small intestines. Cryopreserved tissues have functions similar to those of fresh tissues, as evidenced by RNA-seq in human organoids derived from fresh and cryopreserved tissues, showing similar gene expression patterns (Tsai et al., 2018). However, we did not compare the transcriptomes between fresh and cryopreserved tissues obtained from the same individuals in our study. Further studies are needed to compare the functions of bovine enteroids derived from both tissues. In this study, we resuscitated cryopreserved tissues within 4 mo after cryopreservation. In a human study, tissues stored in liquid nitrogen for 12 mo were revived (Tsai et al., 2018). Further studies are needed to assess how long the tissues can be effectively stored in liquid nitrogen.

Primary and growth medium used in this study was similar to that used in a previous study (Hamilton et al., 2018); however, we were able to culture bovine enteroids with lower inhibitor concentrations, as in a recently published study (Fitzgerald et al., 2019). In these studies, Y-27632 was included in the culture medium during the 7 d of cultivation. However, we showed that Y-27632 was only required during the first 24 h after seeding or passaging as human enteroid cultivation (Tsai et al., 2018). In other studies, the L-WRN (CRL-3276) cell line was used to obtain the recombinant protein to develop enteroids (Powell and Behnke, 2017), and CHIR99021 and A8301 were used instead of LY-2157299 in culture medium (Derricott et al., 2019). Therefore, our method developed bovine enteroids at a lower cost or with fewer steps compared with previous bovine enteroid studies.

A previous human organoid study showed that organoids derived from fresh tissues had greater size compared with organoids derived from cryopreserved tissues at passage 0 (Tsai et al., 2018). Because of these sampling limitations, we could not develop enteroids derived from fresh and cryopreserved tissues from the same individuals. Further studies are needed to compare fresh and cryopreserved tissues obtained from the same individual to understand the factors affecting enteroid proliferation. Interestingly, samples transported on ice for up to 1 h before cryopreservation were successfully resuscitated for enteroid development. This suggests that the early cooling of tissues may be the key to successful cryopreservation. It would also be interesting to know how many hours tissues can be kept on ice before cryopreservation.

In conclusion, a traditional cell cryopreservation solution (i.e., 90% FBS and 10% DMSO) was adequate for tissue cryopreservation, and thereafter, enteroid development. Further studies are needed to understand the limitations of enteroids derived from cryopreserved tissue. This tissue cryopreservation method allows researchers to investigate intestinal function and health (e.g., nutrient uptake and barrier integrity) to develop and use enteroids derived from tissues collected from bovine cattle, even if far distances from the laboratory preclude immediate crypt isolation.
